# Syndecan-3 is selectively pro-inflammatory in the joint and contributes to antigen-induced arthritis in mice

**DOI:** 10.1186/ar4610

**Published:** 2014-07-11

**Authors:** Oksana Kehoe, Neena Kalia, Sophie King, Andrew Eustace, Charlotte Boyes, Ofer Reizes, Anwen Williams, Angela Patterson, Jim Middleton

**Affiliations:** 1 Leopold Muller Arthritis Research Centre, Medical School, ISTM, Keele University, RJAH Orthopaedic Hospital, Oswestry SY10 7AG, UK; 2 Centre for Cardiovascular Sciences, College of Medical and Dental Sciences, University of Birmingham, Birmingham B15 2TT, UK; 3 Faculty of Medicine and Dentistry, School of Oral and Dental Sciences, Lower Maudlin Street, University of Bristol, Bristol BS1 2LY, UK; 4 Lerner Research Institute, 9500 Euclid Avenue, Cleveland OH 44195, USA; 5 Department of Rheumatology, University of Wales College of Medicine, Heath Park, Cardiff CF14 4YS, UK; 6 Rowett Institute of Nutrition and Health, University of Aberdeen, Greenburn Road, Aberdeen AB21 9SB, UK

## Abstract

**Introduction:**

Syndecans are heparan sulphate proteoglycans expressed by endothelial cells. Syndecan-3 is expressed by synovial endothelial cells of rheumatoid arthritis (RA) patients where it binds chemokines, suggesting a role in leukocyte trafficking. The objective of the current study was to examine the function of syndecan-3 in joint inflammation by genetic deletion in mice and compare with other tissues.

**Methods:**

Chemokine C-X-C ligand 1 (CXCL1) was injected in the joints of syndecan-3−/−and wild-type mice and antigen-induced arthritis performed. For comparison chemokine was administered in the skin and cremaster muscle. Intravital microscopy was performed in the cremaster muscle.

**Results:**

Administration of CXCL1 in knee joints of syndecan-3−/−mice resulted in reduced neutrophil accumulation compared to wild type. This was associated with diminished presence of CXCL1 at the luminal surface of synovial endothelial cells where this chemokine clustered and bound to heparan sulphate. Furthermore, in the arthritis model syndecan-3 deletion led to reduced joint swelling, leukocyte accumulation, cartilage degradation and overall disease severity. Conversely, CXCL1 administration in the skin of syndecan-3 null mice provoked increased neutrophil recruitment and was associated with elevated luminal expression of E-selectin by dermal endothelial cells. Similarly in the cremaster, intravital microscopy showed increased numbers of leukocytes adhering and rolling in venules in syndecan-3−/−mice in response to CXCL1 or tumour necrosis factor alpha.

**Conclusions:**

This study shows a novel role for syndecan-3 in inflammation. In the joint it is selectively pro-inflammatory, functioning in endothelial chemokine presentation and leukocyte recruitment and cartilage damage in an RA model. Conversely, in skin and cremaster it is anti-inflammatory.

## Introduction

Syndecans (sdcs) are heparan sulphate proteoglycans (HSPG) composed of a core protein to which heparan sulphate (HS) glycosaminoglycan chains are covalently attached. These molecules form part of the glycocalyx, which comprises a network of membrane-bound proteoglycans and glycoproteins at the cell surface of endothelial cells [1-3]. There are four mammalian syndecans, designated syndecan-1 (sdc-1), -2, -3, and -4, which have protein cores with characteristic structural domains [4,5]. The variable ectodomain, which is exposed to the extracellular environment, contains three to five HS and in some cases chondroitin sulphate chains, and is attached to the cell membrane via a hydrophobic transmembrane segment [6,7]. In addition, there is an intracellular domain containing peptide sequences, which serve as substrates for cellular kinases, enabling syndecans to act as signaling molecules [8].

HSPGs have been shown to play a pro-inflammatory role [9-11]. For example, on endothelial cells they bind and present chemokines to blood leukocytes that leads to leukocyte integrin activation, crawling on the endothelial cell surface and extravasation [12-15]. This interaction involves chemokine immobilisation and concentration at the endothelial surface and stimulation of leukocyte migration into the tissue [16]. Evidence also suggests that HS functions in chemokine transcytosis, which relays chemokines from basal to luminal surfaces of endothelial cells for their presentation to blood leukocytes [12,17-19]. Furthermore, endothelial HS may act as an adhesion molecule, for example binding L-selectin during neutrophil rolling [19]. In contrast, data also indicate that HSPGs may be anti-inflammatory, for example in disease models of nephritis and lung inflammation using sdc-1 and sdc-4 knockout mice [20-25]. Furthermore, removal of HS by heparanase leads to increased leukocyte adhesion to the cremaster endothelium by intravital microscopy, suggesting an anti-inflammatory function [26]. Further work is needed to address the apparent contradictory roles of HSPGs in inflammation. Whether sdcs are pro- or anti-inflammatory may relate to the particular tissue where they are expressed or the inflammatory state.

Inflammation is a central feature of rheumatoid arthritis (RA) that affects around 1% of the population and can result in disability and morbidity. In RA, inflammation of the joint synovium is characterised by the infiltration and activation of leukocytes, which can lead to progressive destruction of cartilage and bone. Chemokines are involved in stimulating the infiltration of leukocytes into inflamed tissue and there is substantial evidence showing an involvement of these mediators and their receptors in RA [27]. For example, chemokine C-X-C ligand 1 (CXCL1) and CXCL8 are abundant in the sera, synovial fluid and synovium in human RA [27-32]. They are produced by synovial macrophages and other cells and attract neutrophils primarily. Furthermore, sdcs have been shown to be expressed in arthritic joints and sdc-4 functions in joint destruction [33-36].

A CXCL8 binding site on endothelial HSPG has been demonstrated in the synovium of RA patients [33]. In order to clarify which HSPG bound the chemokine, immunolocalisation of syndecans and glypicans revealed particularly strong expression of sdc-3 on RA synovial endothelial cells with quantitative PCR confirming endothelial expression. Furthermore anti-sdc-3 antibody and heparanase reduced CXCL8 binding to the endothelium. These data suggest a role for sdc-3 in synovial inflammation. Sdc-3 is the predominant syndecan in the nervous system, where it was first identified, and has been associated with the control of feeding behaviour and the generation of cerebellar fibrillar plaques in Alzheimer’s disease [37,38]. Sdc-3 is also an HSPG of the musculoskeletal system. It has been found in the synovium in adult human joints [33] and is expressed by chondrocytes [39,40]. In addition, sdc-3 is involved in limb morphogenesis and skeletal development and regeneration [41,42]. Several studies have shown that it is expressed by endothelial cells in the synovium, lymph nodes and liver [33,43,44].

Nothing is currently known about the role of sdc-3 in inflammation, unlike sdc-1 and -4 [20-25]. The expression of a CXCL8 binding site on endothelial sdc-3 in human RA suggests a role for this HSPG in inflammatory disease [33] although *in vivo* studies are needed to substantiate this hypothesis. The current study addresses this question, whether genetic deletion of sdc-3 in mice alters leukocyte trafficking in response to murine CXCL1. This chemokine is the functional homologue to CXCL8, which is absent in rodents. The study also addresses if deletion of sdc-3 alters the severity and progression of disease in an RA model. The involvement of sdc-3 in leukocyte recruitment in the synovium was compared to that in the skin and cremaster muscle. This was to find out if sdc can play a different role in different tissues, which may help explain its apparent contradictory function in inflammation. We show that sdc-3 plays a dual role in inflammation depending on the tissue and vascular bed. In the joint it is pro-inflammatory, since its deletion leads to reduced leukocyte recruitment and the severity of arthritis. However, in the skin and cremaster it is anti-inflammatory, since its deletion leads to enhanced leukocyte interaction with the endothelium and recruitment. This is the first study to show a role for sdc-3 in inflammation and reveals its function is tissue-selective.

## Methods

### Animals

Experiments were undertaken in 7- to 10-week-old inbred C57Bl/6 wild-type (sdc-3+/+) and sdc-3 null (sdc-3−/−) mice. Sdc-3−/−mice were generated by Dr Ofer Reizes, Cleveland, USA [37]; they are viable, fertile and develop normally. Procedures were performed with ethical approval from the Home Office, UK, project licence PPL 40/3047.

### Chemokine-driven leukocyte migration into the skin and joints

Mice were injected intradermally or intra-articularly in the knee joint space with recombinant murine CXCL1 (KC) (PeproTech, London, UK) 3 μg/site in phosphate-buffered saline (PBS) [45]. PBS administration was used as a control. After four hours, the animals were sacrificed and skin biopsies or joints were processed for light microscopy. Leukocyte recruitment into the dermis and synovium was observed by light microscopy, neutrophils being identified by their lobed nuclear morphology. To quantitate leukocyte recruitment the number of neutrophils in the synovium was randomly counted in 10 fields of view at x780 magnification per section from sdc-3−/− (n = 8) and sdc-3+/+ (n = 8) mice.

### Myeloperoxidase (MPO) assay

The MPO assay was used as a surrogate marker for the presence of neutrophils in skin tissue and was carried out as described [23]. Briefly, excised pieces of skin from mice were snap frozen in liquid nitrogen and homogenized on ice in 500 μl of PBS with 0.01 M EDTA and a proteinase inhibitor mix (Sigma-Aldrich, Poole, UK) and 1 ml of 1.5% Triton X-100 in PBS. Samples were placed on a rotary shaker at 300 rpm on ice for 30 min, centrifuged at 12,000 × g for 10 min, and supernatants were collected. Total protein concentration for each sample was quantified by BCA Lowry assay (Thermo Scientific Pierce, Cramlington, UK). The protein concentration in all tissue extracts was adjusted to 0.9 mg/ml. MPO activity was determined by using the EnzChek MPO Activity Assay Kit (Invitrogen, Paisley, UK) according to the manufacturer’s instructions.

### Immunofluorescence

For CXCL1 (KC) and E-selectin detection in skin and joint samples we used a tyramide signal amplification kit [46] (Molecular Probes, Invitrogen). Briefly, formalin-fixed, wax-embedded sections of skin and joints were de-waxed, rehydrated, washed in PBS, and skin subjected to antigen retrieval in Tris-HCl buffer, pH 9.0, at 100°C in a water-bath for 20 min; for joints antigen retrieval was in 10 mM Tris-HCl buffer, 1 mM EDTA and 0.05% Tween 20, pH 9.0, overnight at 65°C. The endogenous peroxidase was blocked by incubation for 10 min with 3% H_2_O_2_ followed by incubation with 1% blocking reagent for 60 min at room temperature. Sections were incubated for 60 min with rabbit anti-murine CXCL1 polyclonal antibody (PeproTech) at 2 μg/ml or rat anti-murine E-selectin monoclonal antibody at 5 μg/ml (kindly supplied by Dr Alexander Zarbock, University of Munster, Germany). Sections were then treated with HRP-conjugated goat anti-rabbit or goat anti-rat secondary antibodies for 60 min, then Alexa Fluor™ 488 tyramide for 10 min. For sdc immunofluorescence, sections were treated with affinity purified rabbit anti-mouse sdc-3 (1:500) [37] goat anti-rabbit Alexa 594 antibody (Invitrogen) containing 10% mouse serum. Tissue sections were stained with DAPI for cell nuclei and analysed using a Leica IX51 microscope (Leica, Wetzlar, Germany). Control sections were negative when treated with rabbit or rat immunoglobulin (Ig)G instead of primary antibodies (added at the same concentrations) or when the primary antibodies were omitted.

For dual labelling, sections were treated with anti-E-selectin (as above) together with rabbit anti-von Willebrand factor (1:100; Dako, Ely, UK) followed by goat anti-rabbit Alexa 594 second antibody (Invitrogen) For quantitation of E-selectin expression, five vessels were randomly sampled per section of synovium (n = 6 sdc-3 null and wild-type mice with antigen-induced arthritis (AIA) at day 3) and skin (n = 8 sdc-3 null and wild-type mice). The numbers of vessels showing a luminal E-selectin distribution were counted. For quantitation of CXCL1 on endothelial cells five vessels were randomly sampled per skin (n = 8 sdc-3+/+and n = 9 sdc-3−/−mice) and joint (n = 6 sdc-3+/+and sdc-3−/−mice) section.

Heparanase treatment of sections was performed using a previously described method [33]. Briefly, formalin-fixed, wax-embedded sections of CXCL1-injected joints were de-waxed, rehydrated, washed in PBS, and subjected to antigen retrieval as above. The sections were treated with 20 units/ml of heparanase I and 4 units/ml heparanase III (both Sigma-Aldrich, UK) in HBSS, or HBSS alone, for 1.5 hours at 37°C. After enzymatic treatment, the samples were rinsed twice with HBSS before CXCL1 immunolocalisation as described above.

### Intravital microscopy

The effects of sdc-3−/−on leukocyte rolling and stationary adhesion was also measured *in vivo* in the cremaster muscle microcirculation using intravital microscopy (PPL 40/2747) [47]. Briefly, in anaesthetized (ketamine/xylazine; intraperitoneally (ip)) mice, the testis was exposed through a small scrotal incision and the cremaster muscle exteriorised, cleared of connective tissue and pinned across a glass coverslip on a specialised microscope stage. The muscle was continuously superfused with bicarbonate-buffered saline (131.7 mM NaCl, 4.69 mM KCl, 2.7 mM CaCl_2_, 2.1 mM MgCl_2_ and 14.44 mM NaHCO_3,_ pH 7.4), equilibrated with 5% CO_2_ in N_2_ and maintained at 37°C. Prior to intravital observations, mice were either pre-treated with an intrascrotal injection of TNFα (500 ng in 200 μl; R&D Systems, Abingdon, UK) for three hours or the cremaster was superfused with CXCL1 (5nM in 500 ml; Peprotech) for 1 hour. Control mice received a PBS vehicle. Leukocyte-endothelial cell interactions were observed in single unbranched post-capillary venules (PCV; 20 to 50 μm diameter). Leukocyte rolling was determined by counting numbers of cells rolling along a 100 μm PCV segment within 60 seconds. A leukocyte was considered firmly adherent if it remained stationary for ≥30 seconds.

### Induction of murine antigen-induced arthritis (AIA)

Experiments were performed in 7- to 8-week-old male mice. Murine AIA was induced as described [48]. Briefly, mice were immunised subcutaneously with 1 mg/ml of methylated bovine serum albumin (mBSA) emulsified with an equal volume of Freund’s complete adjuvant and injected intraperitoneally with 100 μl heat-inactivated *Bordetella pertussis* toxin (all reagents from Sigma-Aldrich). The immune response was boosted one week later. Twenty-one days after the initial immunisation, murine AIA was induced by intra-articular injection of 10 mg/ml mBSA in PBS in the right knee (stifle) joint. For a control, the same volume of PBS was injected into the left knee joint.

Animals were inspected daily for arthritis development by measuring knee joint diameters using a digital micrometer. The difference in joint diameter between the arthritic (right) and non-arthritic control (left) in each animal gave a quantitative measure of swelling (in mm).

### Histological assessment

Animals were killed at the indicated times after induction of arthritis. Joints were fixed in neutral buffered formal saline, and decalcified with formic acid at 4°C before embedding in paraffin. Mid-sagittal serial sections (7 μm thickness) were cut and stained with haematoxylin and eosin (H&E). Two independent observers blinded to the experimental groups scored sections. Synovial hyperplasia, cellular exudate and cartilage depletion were scored from 0 (normal) to 3 (severe); synovial infiltrate was scored from 0 to 5 [48,49]. Cartilage damage was scored on serial haematoxylin/safranin O-stained sections. All parameters were subsequently summed to give an arthritis index (mean ± SEM).

### Statistics

Differences between groups were compared by Mann-Whitney *U* or unpaired *t* tests, with *P* <0.05 being deemed as significant.

## Results

### Sdc-3 deletion reduces neutrophil recruitment in CXCL1-injected joints

**Figure 1:**
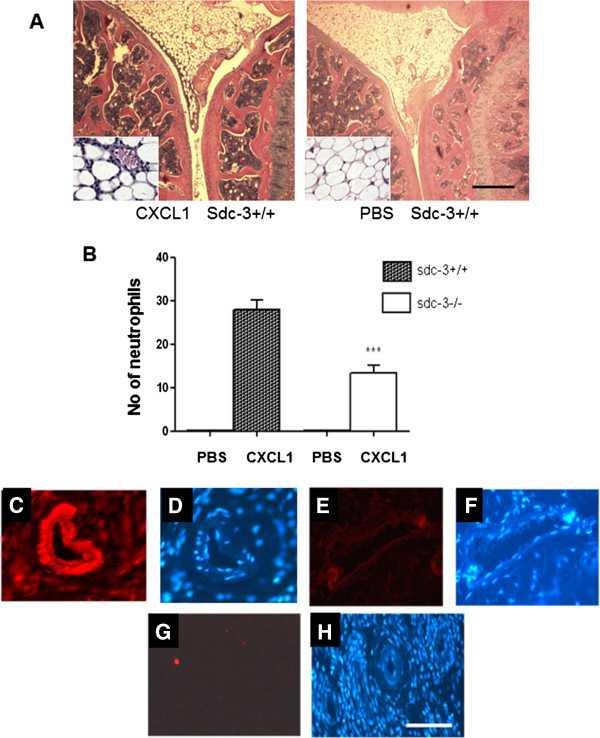
**Sdc-3 deletion reduces neutrophil recruitment in CXCL1-injected joints.** Sdc-3 null and wild-type mice were injected intra-articularly with 3 μg per knee joint of recombinant murine CXCL1 or PBS and after 4 hours processed for histology. **(A)** Haematoxylin and eosin staining reveals neutrophil recruitment in the synovium following CXCL1 administration with PBS injected as control; insets show detail of synovium. Results are shown from a wild-type joint. Scale bar 500 μm (for inserts 50 μm). **(B)** Decrease in the number of recruited neutrophils in sdc-3−/−mice compared to wild-type animals following CXCL1 administration. Data are means ± SEM, n = 8 mice per treatment. ^***^P <0.0001 comparing CXCL1-injected joints. **(C)** Sdc-3 immunolocalises to the blood vessel in synovium of wild-type mice. **(D)** is the same image as **(C)** stained for DAPI to show cell nuclei. **(E)** absent staining for sdc-3 in a blood vessel in sdc-3 −/−synovium. **(F)** is the same image as **(E)** stained for DAPI to show cell nuclei. **(G)** is a negative control of the synovium of wild-type mouse in the absence of sdc-3 antibody and **(H)** is the same area stained for DAPI to show the presence of blood vessels. Bar =120 μm in C to H. CXCL1, chemokine C-X-C ligand 1; DAPI, 4',6-diamidino-2-phenylindole; PBS, phosphate-buffered saline.

To examine the effects of sdc-3 on inflammation we first studied chemokine-driven leukocyte migration into the knee joint. Intra-articular injection of murine CXCL1 stimulated the influx of neutrophils into the synovium of the joints of sdc-3−/−and wild-type mice (Figure 1A), whereas PBS-injected controls were negative for neutrophils. To compare and quantitate leukocyte recruitment the number of migrated neutrophils in the synovia was counted. A significant decrease (*P* <0.0001; *t* test) in the number of neutrophils recruited in sdc-3−/−mice was observed compared to wild type after CXCL1 injection (Figure 1B). In PBS-injected controls there was no neutrophil recruitment in synovia of sdc-3−/−and sdc-3+/+mice.

### Reduced chemokine presentation by synovial endothelial cells in sdc-3−/−mice

**Figure 2:**
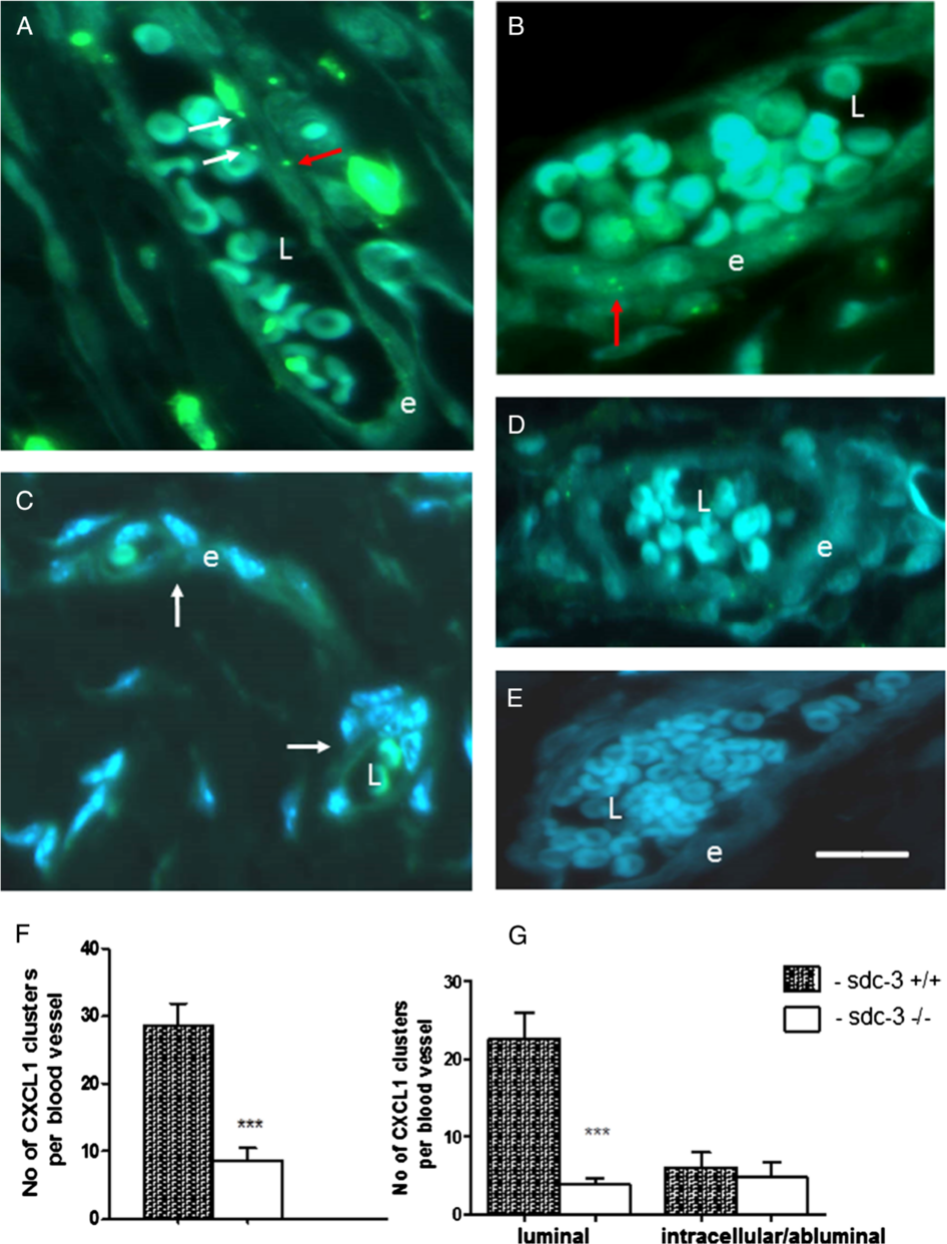
**Reduced chemokine presentation by synovial endothelial cells in sdc-3−/−mice.** In the same samples as in Figure 1 sections of CXCL1-injected joints of wild-type and sdc-3−/−mice were stained with a CXCL1 antibody using tyramide amplification, and DAPI, and viewed by immunofluorescence. **(A and B)** Synovial endothelial cells of wild-type mouse joint; CXCL1 occurs as clusters with white arrows showing examples of luminal chemokine and red arrows intracellular or abluminal chemokine. The endothelial cell layer is labelled (e) and the lumen of the blood vessel (L) containing red blood cells. **(C)** No CXCL1 clusters are present in the endothelial cells of PBS-injected controls (arrows); this image is from a wild-type mouse. **(D)** There are less CXCL1 clusters in endothelial cells of following treatment with heparanase I and III to degrade heparan sulphate prior to immunostaining. **(E)** is a negative control of the synovium of wild-type mouse in the absence of CXCL1 antibody. Bar = 30 μm in A to E. **(F)** Quantification of CXCL1 staining shows decrease in the numbers of endothelial CXCL1 clusters per blood vessel in sdc-3−/− (n = 6) compared to wild-type (n = 6) joints. Data are mean ± SEM. ^***^P = 0.0003. **(G)** shows the data in **(F)** expressed as number of CXCL1 clusters at the luminal surface or intracellularly/abluminally in synovial endothelial cells. There is a reduction of the numbers of luminal CXCL1 clusters in sdc-3−/−mice compared to wild type, ^***^P = 0.0002. Data are means ± SEM. CXCL1, chemokine C-X-C ligand 1; DAPI, 4',6-diamidino-2-phenylindole; PBS, phosphate-buffered saline.

Experiments were performed to examine if murine synovial endothelial cells expressed sdc-3, similar to human synovial endothelial cells [33]. Immunoreactive sdc-3 was demonstrated in blood vessels in normal and AIA synovia of wild-type mice but not in sdc-3 null mice as control (Figure 1C-F). Further controls in the absence of sdc-3 antibody (Figure 1G and H), or when substituted with rabbit control Ig, were also negative. Chemokines may be produced extravascularly and a transcytosis mechanism allows for these chemokines to be transported to the luminal surface of the endothelium [12,45,50]. At this interface HS is then involved in presenting the bound chemokines to signalling receptors on the surface of blood leukocytes [51]. We wanted to test if deletion of sdc-3 would alter chemokine binding and presentation at the endothelial surface, which may explain the reduced neutrophil recruitment in sdc-3 null mice in response to CXCL1 (Figure 1B). Sections of the same CXCL1-injected joints of wild type (n = 8) and sdc-3−/− (n = 9) mice as used in Figure 1 were immunostained with a CXCL1 antibody using tyramide amplification and observed by confocal immunofluorescence. CXCL1 appeared as discrete clusters associated with synovial endothelial cells of sdc-3 null and wild-type joints (Figure 2A and B). In PBS-injected control joints there were no CXCL1 clusters in synovial endothelial cells of knockout and wild-type mice (Figure 2C). Quantification revealed a three-fold reduction in the numbers of endothelial CXCL1 clusters per blood vessel in sdc-3−/−compared to sdc-3+/+joints (*P* = 0.0003; *t* test) (Figure 2F). These data were further separated into luminal or intracellular/abluminal distribution of endothelial CXCL1. The numbers of luminal CXCL1 clusters were reduced 6-fold in sdc-3−/−mice compared to wild type (*P* = 0.0002; *t* test) (Figure 2G). However, there was no significant difference comparing intracellular/abluminal numbers of CXCL1 clusters in sdc-3 null and wild-type mice (Figure 2G).

Serial sections of CXCL1-injected joints were treated with heparanase I and III to degrade heparan sulphate prior to CXCL1 immunolocalisation. Use of these enzymes resulted in lack of CXCL1 immunofluorescence in endothelial cells of wild-type and sdc-3−/−synovial blood vessels (Figure 2D). Quantitation revealed that for wild type the mean number of endothelial CXCL1 clusters per blood vessel after heparanase digestion was 2.8 ± 0.7 (mean ± SE, n = 6), which was significantly lower than without heparanase (28.7 ± 3.2, see Figure 2F) (*P* <0.0001; *t* test). For sdc-3 null mice the mean number of endothelial CXCL1 clusters per blood vessel after heparanase was 2.7 ± 1.8 and 8.7 ± 1.8 without heparanase (both mean ± SE, n = 5) (Figure 2F) and these values did not significantly differ. These heparanase data suggest that the heparan sulphate chains of endothelial sdc-3 bind CXCL1 clusters. Controls in the absence of anti-CXCL1 were negative (Figure 2E). Sections were also immunostained for E-selectin. Although E-selectin was detected in synovial endothelial cells, it was less abundant than in skin endothelial cells, and there was no significant difference in E-selectin distribution between sdc-3−/−and sdc-3+/+mice in the presence or absence of CXCL1 (data not shown).

### Less severe AIA in sdc-3−/−mice

**Figure 3:**
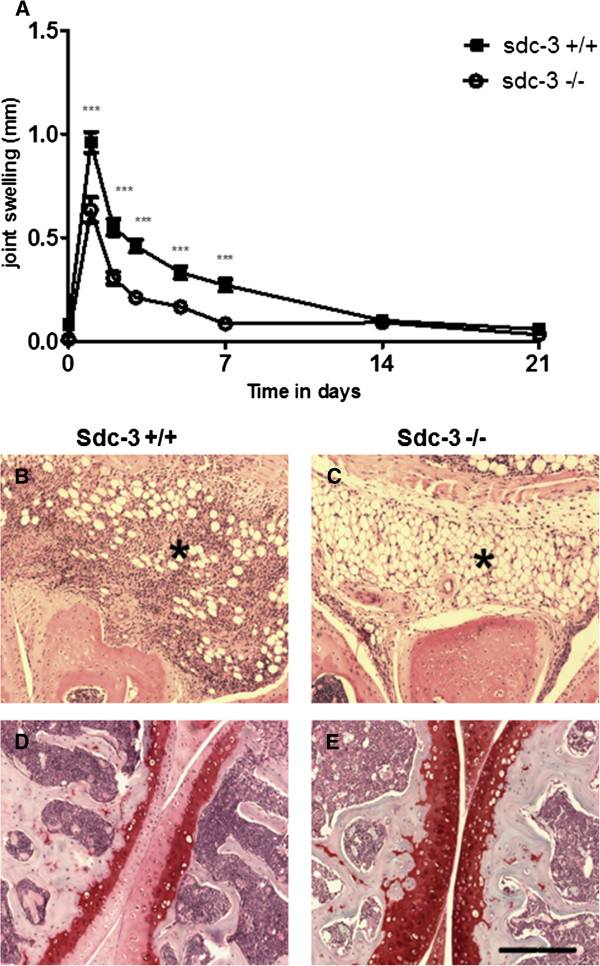
**Antigen-induced arthritis in sdc-3−/−and wild-type mice.** Arthritis was induced by intra-articular injection of methylated BSA in the right knee (stifle) joint. For a control, PBS was injected into the left knee joint. **(A)** Joint swelling after arthritis induction at t = 0, n ≥16 sdc-3+/+and sdc-3−/−mice per time point. Data are means ± SEM for right knee after subtraction of left knee control. ^***^P <0.001 comparing sdc-3−/−and sdc-3+/+mice at the time points indicated. **(B-E)** Synovial inflammation and cartilage destruction in mouse antigen-induced arthritis three days after induction. **B**, wild-type knee joint with marked leukocyte infiltration of the synovium (^*^), mainly neutrophils. **D**, wild-type knee joint with loss of proteoglycan staining in the surface regions of tibial and femoral articular cartilage. **C** and **E** show sdc-3−/−knee joints and lack of leukocyte infiltration (^*^) in the synovium and degradation of articular cartilage. **B** and **C** were stained with haematoxylin and eosin and **D** and **E** with haematoxylin and safranin-O. Scale bar =160 μm in **B** to **E**. BSA, bovine serum albumin; PBS phosphate-buffered saline.

Since the above data suggested that sdc-3 is pro-inflammatory in the joint the role of this HSPG in a model of inflammatory disease, namely RA, was assessed. AIA was induced in the knee joints of sdc-3−/−and wild-type mice and joint swelling, synovial inflammation and cartilage destruction were measured. Knee joint diameter (swelling), a clinical indication of joint inflammation, was significantly less in sdc-3−/−mice compared to wild type 24 hours after arthritis induction (0.63 ± 0.06 mm versus 0.96 ± 0.05 mm; *P* <0.0001 ANOVA and Tukey post tests) (Figure 3A). This difference continued for approximately seven days post-intra-articular mBSA administration (*P* <0.001).

Histologically, AIA was characterised by synovial hyperplasia of the synovial lining layer, infiltration of the synovial sublining by leukocytes, exudate in the joint cavity, and loss of proteoglycan from the articular cartilage, as observed in haematoxylin/eosin and haematoxylin/safranin-O stained sections (Figure 3B and D). These changes did not occur in contralateral knee joints, which were injected with PBS instead of mBSA and appeared histologically normal. The degree of leukocyte infiltration and cartilage destruction (proteoglycan loss) appeared less severe in sdc-3 null mice compared to wild type (Figure 3B versus C, D versus E). In order to quantitate these changes, parameters were scored as a measure of disease severity and differences between sdc-3 null and wild-type mice were apparent (Table 1). There was a significant reduction of synovial leukocyte infiltrate comprising mainly neutrophils (*P* <0.01), cartilage depletion (*P* <0.05) and arthritic index representing overall disease severity (*P* <0.01) in sdc-3−/−compared to sdc-3+/+mice at day 3 (Table 1) (all comparisons Mann-Whitney test). At day 14 and 21 post intra-articular injection of mBSA, there were no significant differences between wild type and sdc-3−/−for all parameters except exudate, which was significantly reduced in sdc-3 null mice (*P* <0.02 Mann-Whitney test) at day 21. In addition, the infiltrate was less in sdc-3−/−mice compared to sdc-3+/+although this approached significance at day 21 (*P* = 0.06 Mann-Whitney test).

**Table 1:** Joint inflammation and cartilage damage on day 3 of antigen-induced arthritis

**Species**	**Hyperplasia**	**Synovial infiltrate**	**Exudate**	**Cartilage depletion**	**Arthritis index**
Syndecan 3−/−	2.07 ± 0.23	2.67 ± 0.67^**^	0.63 ± 0.24	0.63 ± 0.24^*^	6.00 ± 0.75^**^
Syndecan-3+/+	2.57 ± 0.15	3.97 ± 0.24	1.40 ± 0.31	1.40 ± 0.27	9.33 ± 0.74

Synovial hyperplasia of the lining layer, synovial infiltration of the sublining by leukocytes, exudate in the joint cavity, and loss of proteoglycan from the articular cartilage were observed in haematoxylin/eosin and haematoxylin/safrinin-O-stained sections. Sections were scored blind by two independent observers from 0 to 3 (hyperplasia, exudate and cartilage depletion) or 0 to 5 (infiltrate). The arthritis index is the sum of all observations. ^*^P <0.05 and ^**^P <0.01 of the parameter compared to wild-type mice using Mann-Whitney *U* test.

### Sdc-3 deletion provokes enhanced neutrophil recruitment in CXCL1-injected skin

**Figure 4:**
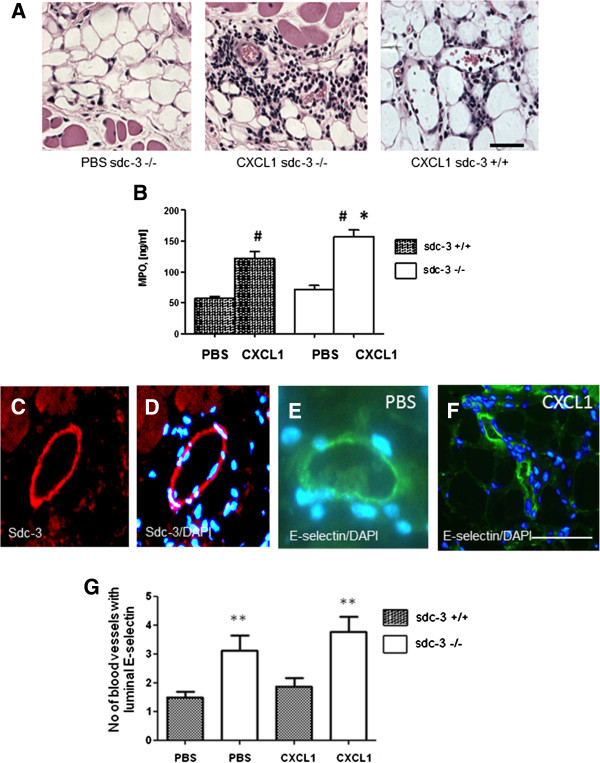
**Leukocyte migration after intradermal injection of CXCL1 in sdc-3 null and wild-type mice and altered distribution of E-selectin. (A)** Mice were injected intradermally with recombinant murine CXCL1 (3 μg/site in PBS) or PBS as control. After four hours the animals were sacrificed and skin biopsies were processed for light microscopy. Haematoxylin and eosin staining of skin sections show neutrophil recruitment into the dermis in sdc-3−/− (mid panel) and sdc-3+/+ (right panel) mice. The left micrograph shows skin from an sdc-3−/−mouse following PBS administration. Scale bar 50 μm. **(B)** Using the same samples as in **(A)** lysates were prepared from skin biopsies and myeloperoxidase (MPO) activity measured as a marker for the presence of neutrophils. Increased MPO activity is observed in sdc-3−/−compared to sdc-3+/+skin tissue. For sdc-3−/−mice n = 9 with CXCL1 and with PBS, and for sdc-3+/+mice n = 10 with CXCL1 and with PBS. Data are means ± SEM. ^*^P <0.03 compared with CXCL1 injected sdc-3+/+mice. ^#^*P* <0.005 compared to respective PBS control. **(C and D)** Sdc-3 staining of endothelial cells is shown in a dermal venule from a wild-type mouse. **(E and F)** E-selectin shows a luminal distribution in the endothelial cells of the dermis after PBS **(E)** and CXCL1 **(F)** administration, this section is from a sdc-3−/−mouse. Scale bar 50 μm for E-selectin and 25 μm for sdc-3 micrographs. **(G)** Quantification of the number of blood vessels with a luminal E-selectin distribution in the dermis of sdc-3+/+ (n = 8) compared to sdc-3−/− (n = 9) mice. Data are mean ± SEM, ^**^P <0.008 compared to sdc-3 wild-type mice. CXCL1, chemokine C-X-C ligand 1; PBS, phosphate-buffered saline.

In order to compare the inflammatory role of sdc-3 in the joint with other tissues, skin was injected intradermally with murine CXCL1 or PBS as control (as Figure 1). Four hours after CXCL1 injection, histological staining revealed an influx of leukocytes into the dermis in sdc-3 null and wild-type mice (Figure 4A). These leukocytes were identified histologically as being neutrophils. To quantitate differences in neutrophil recruitment, we examined MPO activity as a marker for the presence of these cells in skin extracts. There was an increase in MPO levels following CXCL1 injection compared to PBS in sdc-3 null (*P* <0.005; *t* test) and wild-type (*P* <0.005; *t* test) mice (Figure 4B). Interestingly, a significant 30% increase (*P* <0.03; *t* test) in MPO activity over wild type was observed in sdc-3−/−mice after CXCL1 administration (Figure 4B). Baseline activity of MPO in PBS-injected control samples did not significantly differ between sdc-3 null and wild-type mice.

Immunofluorescence using anti-murine sdc-3 showed that this HSPG was expressed in the endothelium of the dermis in wild-type mice (Figure 4C and D). To further investigate the potential mechanism of increased neutrophil recruitment after CXCL1 challenge adhesion molecule expression was examined. E-selectin immunolocalisation was performed in skin tissue sections (Figure 4E and F). This adhesion molecule is expressed by dermal endothelial cells and is involved in the rolling stage of leukocyte adhesion to the endothelium [52-55]. Using dual labelling E-selectin co-localised with von Willebrand factor as a marker of endothelial cells (Additional file 1C to E), and the proportion of von Willebrand factor positive dermal blood vessels that expressed E-selectin was >95% (n >15 vessels per wild-type and sdc-3−/−mouse). E-selectin exhibited a predominantly luminal or intracellular distribution in the endothelial cells of the dermis in sdc-3−/−and sdc-3+/+mice (Figure 4E and F; Additional file 1A and B). Quantification revealed that there was a two-fold increase in the numbers of vessels with a luminal E-selectin distribution in sdc-3−/−mice compared to wild type (*P* <0.008 Mann-Whitney test) following CXCL1 administration (Figure 4G). When PBS was administered instead of CXCL1, as vehicle-injected control, there was also significantly more vessels with a luminal E-selectin distribution in sdc-3 null mice compared to wild type (Figure 4G). In sdc-3−/−mice there was no significant difference in luminal E-selectin between CXCL1- and PBS-injected skin (Figure 4G), suggesting that this chemokine was not affecting E-selectin distribution. After injection of CXCL1, this chemokine could be detected as a uniform distribution in endothelial cells of dermal venules by immunofluorescence, however, there was no significant difference in the number or percentage of these cells positive for CXCL1 in sdc-3−/− (n = 8) and wild-type (n = 9) mice (data not shown); this suggests that CXCL1 presentation in skin may be occurring by a different proteoglycan than sdc-3. Control sections treated in the absence of E-selectin, von Willebrand or CXCL1 antibodies were negative.

### Increased rolling and adhesion of leukocytes in cremaster venules in sdc-3 null mice

**Figure 5:**
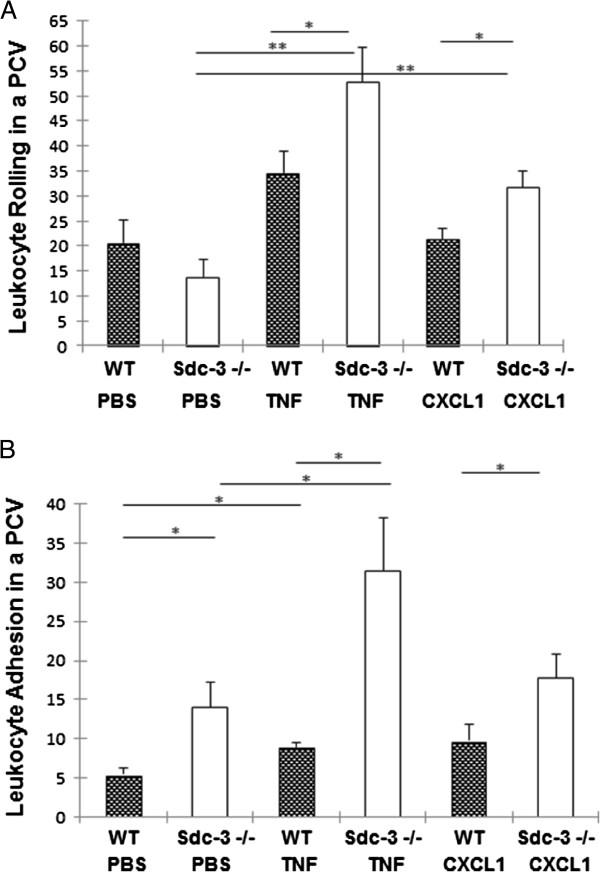
**Increased rolling and adhesion of leukocytes in cremaster muscle post-capillary venules (PCV) in sdc-3 null mice by intravital microscopy. (A)** Significant increases in leukocyte rolling are observed in TNFα-stimulated sdc-3−/−mice when compared to either TNFα-stimulated wild-type (WT) or unstimulated syn-3−/−mice. Similarly, CXCL1 stimulation induces greater rolling in sdc-3−/−mice. **(B)** Similar increases in leukocyte adhesion are observed in TNFα- and CXCL1-stimulated sdc-3−/−mice. Data are means ± SEM, n = 3 to 6 mice per treatment. ^*^P <0.05; ^**^P <0.01. CXCL1, chemokine C-X-C ligand 1; TNFα, tumour necrosis factor alpha.

Intravital microscopy was used to examine the effects of sdc-3 gene deletion on the rolling and firm adhesion of leukocytes to venular endothelial cells in the cremaster muscle. The basal number of rolling leukocytes was not significantly different between unstimulated (PBS-treated) sdc-3−/−mice and wild-type mice (Figure 5A). Although TNFα stimulation increased leukocyte rolling in wild-type mice, this did not reach significance. However, a significant increase in rolling was observed in TNFα-stimulated sdc-3−/−mice when compared to either unstimulated sdc-3−/− (*P* <0.01) or TNFα-stimulated wild-type (*P* <0.05) mice (Figure 5A). Indeed, when compared to unstimulated sdc-3−/−, an almost four-fold increase in rolling was observed. Similarly, CXCL1 stimulation did not increase leukocyte rolling in wild-type mice, but it was associated with a significant increase in rolling in sdc-3−/−mice when compared to unstimulated sdc-3−/− (*P* <0.01) or CXCL1-stimulated wild-type (*P* <0.05) mice (Figure 5A).

Interestingly, the basal number of adherent leukocytes was significantly (*P* <0.05) increased in unstimulated sdc-3−/−mice when compared to wild-type mice, with more than double the numbers of adherent cells observed (Figure 5B). As expected, TNFα stimulation significantly (*P* <0.05) increased leukocyte adhesion in wild-type mice when compared to unstimulated wild-type mice. However, this effect was more dramatic in the sdc-3−/−mice, with significantly increased leukocyte adhesion observed when compared to either unstimulated sdc-3−/− (*P* <0.05) or TNFα-stimulated wild-type (*P* <0.05) mice (Figure 5B). Indeed, when compared to unstimulated sdc-3−/−, a 2.2-fold increase in adhesion was observed. Although CXCL1 stimulation did not increase leukocyte adhesion in wild-type mice, it was associated with a significant increase in adhesion in sdc-3−/−mice when compared to CXCL1-stimulated wild type (*P* <0.05). This did not reach significance when compared to unstimulated sdc-3−/−, presumably reflecting increased basal adhesion in the sdc-3−/− (Figure 5B). All statistical comparisons for intravital microscopy were made by ANOVA followed by Tukey’s pairwise tests.

## Discussion

The current study demonstrated that sdc-3 played a role in inflammation, but interestingly, highlighted both pro- and anti-inflammatory properties for this proteoglycan depending upon the tissue and nature of the inflammatory insult. In the joint, chemokine administration resulted in reduced neutrophil influx in the synovium of sdc-3 null mice indicating that this HSPG is playing a pro-inflammatory role. This effect may be attributed to chemokine presentation by sdc-3 on synovial endothelial cells since deletion of this HSPG reduced the presence of chemokine CXCL1 on these cells. Furthermore, heparanase reduced the amount of endothelial CXCL1 suggesting the involvement of HS chains in binding CXCL1. The chemokine in synovial endothelial cells was not uniformly distributed but appeared to be bound to sdc-3 in clusters. Thus CXCL1 may be concentrated and immobilised into clusters at the endothelial surface for presentation to blood leukocytes. This is in agreement with Hardy *et al*. [15] who found a focal distribution of CCL2 bound to HS at the apical endothelial surface during leukocyte transendothelial migration *in vitro*. CXCL1 clusters were particularly reduced at the endothelial surface in sdc-3−/−mice whereas in the remainder of the cell in intracellular/abluminal locations this was not the case. This suggests that sdc-3 may be particularly involved in chemokine presentation whereas other molecules may play a more dominant role in transcytosis, such as the Duffy antigen/receptor for chemokines [45,56]. The finding of sdc-3 binding and presenting CXCL1 in the current study is in agreement with our previous study [33]. In human RA there is induction of a CXCL8 binding site on sdc-3 HS chains of synovial endothelial cells. Mice lack CXCL8 and CXCL1 is the functional equivalent in the murine system. Therefore taken together, these two studies suggest that sdc-3 may be involved in binding CXC chemokines and stimulating leukocyte trafficking into the RA synovium.

A pro-inflammatory function of sdc-3 was also apparent in a murine model of RA. Induction of AIA in the knee joint resulted in reduced joint swelling in sdc-3 knockout mice suggesting that sdc-3 contributes to the clinical manifestation of the disease. This HSPG is also involved in underlying inflammatory changes such as leukocyte accumulation into the synovium, which was reduced in sdc-3 null mice as was the overall histological severity of disease. The pro-inflammatory function of sdc-3 in AIA may be due to chemokine presentation by synovial endothelial cells. Furthermore a role for sdc-3 in joint damage, which is a major feature of RA, is implicated as shown by the inhibitory effects of sdc-3 deletion on cartilage damage. This involvement of sdc-3 in cartilage damage may be related to its pro-inflammatory function in the synovium, via leukocyte recruitment leading to cytokine or degradative enzyme release. However, the effects of loss of sdc-3 in the arthritis model may be mediated, at least in part, by cells other than endothelial cells, since sdc-3 is also expressed by chondrocytes [39,40]. Further studies involving conditional deletion of sdc-3 in selected cell types and examining the effect on arthritis severity would be of interest in this respect.

Recent data suggest a role for sdc-4 in inflammatory arthritis [34,35]. Using the human TNF transgenic mouse model (hTNFtg) of RA, sdc-4 was involved in the attachment and invasion of synovial fibroblasts into cartilage, contributing to cartilage destruction. Sdc-4 also regulates ADAMTS-5 activation and cartilage breakdown [36]. This suggests that sdcs may be involved in various aspects of joint inflammation and damage in arthritis, with endothelial sdc-3 functioning in leukocyte recruitment and fibroblast sdc-4 in cartilage destruction.

Deletion of sdc-3 in the skin had the opposite effect compared to that in the joint. When CXCL1 was injected into the skin neutrophil recruitment was enhanced in sdc-3−/−mice compared to wild type suggesting that this HSPG plays an anti-inflammatory function in this tissue. The effect may be mediated, at least in part, by the adhesion molecule E-selectin since the luminal distribution of E-selectin increased in knockout animals suggesting increased expression of this adhesion molecule at the endothelial surface. This may lead to elevated neutrophil recruitment in the presence of CXCL1. E-selectin is expressed in normal skin venules where it is upregulated in skin inflammation [52-55]. Sdc-3 is part of the glycocalyx, which can form an anti-adhesive layer to blood leukocytes at the endothelial surface and it has been proposed that this may mask endothelial adhesion molecules inhibiting leukocyte-endothelial interactions [1,10]. Steric hindrance may play a role in this process since the glycocalyx can reach microns in thickness whereas selectins only extend <50 nm from the endothelial surface [1,57]. Therefore loss of sdc-3 in knockout mice may lead to the unmasking or altered expression of E-selectin at the luminal endothelial surface leading to increased leukocyte recruitment. This is in agreement with other studies that show that stimuli that degrade the glycocalyx or induce a more open mesh such as enzymes, cytokines, or ischaemia and reperfusion appear to uncover adhesion molecules, thereby allowing leukocytes to interact with the endothelium [1-3,26]. For example, heparanase, which is a glycosidase that removes HS, causes increased leukocyte adherence at the endothelial surface in the cremaster venules of mice by intravital microscopy [26]. In the present study, endothelial sdc-3 does not appear to be presenting the chemokine CXCL1 in the skin since there was no difference in the presence of this chemokine on dermal venules in wild-type and knockout mice and other HSPGs may be more involved in this mechanism. Thus in the skin sdc-3 may be involved in regulating leukocyte adhesion via altering the distribution or expression of the adhesion molecule E-selectin.

Since the data obtained from the skin demonstrated an anti-inflammatory role for sdc-3, we further investigated its role using the more direct approach of intravital microscopy, which allowed real-time dynamic images of leukocyte adhesion to be monitored in anaesthetised mice *in vivo*. Furthermore, the effects on sdc-3 deletion on leukocyte rolling could also be assessed, which was not possible on static sections. Increased numbers of rolling and adherent leukocytes in the venules of sdc-3−/−mice in response to either CXCL1 or TNFα was observed compared to wild type. These results suggest that sdc-3 has an inhibitory effect on leukocyte-endothelial interactions in response to inflammatory stimuli and are in accord with those in skin. Intravital microscopy has been performed in sdc-1 null mice following TNFα treatment where there is increased adhesion of leukocytes to endothelial cells of the mesentery venules [11,58]. These intravital data, together with ours, indicate that sdc-3 and sdc-1 play similar roles in cremaster and mesenteric venules using similar inflammation models. In these tissues sdc-3 and sdc-1 appear to be negative regulators of leukocyte-endothelial interactions.

The anti-inflammatory role of sdc-3 in our models in skin and cremaster is similar to that of sdc-1 and -4 in inflammatory disease models. Sdc-1 gene deletion in mice reduces inflammation in models of allergic contact dermatitis, allergic lung disease, colitis and nephritis, with increased leukocyte recruitment and more severe disease [20,21,23,24]. Similarly sdc-4 null mice exhibit increased inflammation and neutrophil recruitment in a model of pulmonary inflammation and lung injury [25]. Thus our finding of sdc-3 having a pro-inflammatory role in synovium in a mouse model of RA is more unusual amongst the different sdc knockout models. Whether these HSPGs have pro- or anti-inflammatory functions may depend on the sdc, the tissue or cell-type where they are expressed and/or the type of inflammation. Furthermore, specific targeting of sdcs tailored to particular inflammatory diseases is called for if they are to be exploited therapeutically in human diseases. For example, blocking sdc-3 or sdc-4 in human RA would be of potential interest in reducing inflammation and joint destruction, whereas this strategy may have opposite effects in certain inflammatory conditions of the skin, lung, gut and kidney.

In the current study, sdc-3 was found to be expressed by endothelial cells in murine synovium and skin. This is in agreement with human tissues where endothelial sdc-3 was found particularly expressed in the endothelial cells of RA synovium [33]. Interestingly, sdc-3 is also found in lymphoid tissue, where this HSPG perfectly delineates some of the high endothelial venules [44]. These venules are the preferred sites of lymphocyte extravasation which, taken with the findings of the current study, suggests a role for this HSPGs in lymphocyte trafficking in the lymph nodes. Sdc-3 is also expressed by the endothelial cells in human liver [43].

## Conclusions

Sdc-3 appears to have a tissue-selective role in inflammation being pro-inflammatory in the joint, which may be mediated by endothelial chemokine presentation. It is also involved in leukocyte accumulation and cartilage damage in joints with AIA. In the skin and cremaster it may be anti-inflammatory, contributing to the anti-adhesive properties of the endothelial glycocalyx. This study helps clarify the contradictory roles of HSPGs being reported as pro-and anti-inflammatory and suggests the importance of tissue-dependent functions of endothelial cells in the case of sdc-3. Furthermore, it suggests that targeting sdc-3 in the joint in inflammatory arthritis would be a therapeutic strategy.

## Abbreviations

AIA: antigen-induced arthritis; CXCL1: chemokine C-X-C ligand 1; HSPG: heparan sulphate proteoglycan; Ig: immunoglobulin; mBSA: methylated bovine serum albumin; MPO: myeloperoxidase; PBS: phosphate-buffered saline; PCV: post-capillary venule; RA: rheumatoid arthritis; sdc: syndecan; TNF: tumour necrosis factor.

## Competing interests

The authors declare that they have no competing interests.

## Authors’ contributions

OK performed most of the experimental work, interpreted the data and drafted the manuscript. NK performed the intravital microscopy, interpreted the data and contributed to writing the manuscript. SK, AE and CB performed the immunofluorescence microscopy and data interpretation. OR developed and provided the null mice and helped design the study. AW and AP interpreted the data and helped design the study. JM designed the study, interpreted data and wrote the manuscript. All authors read and approved the manuscript.

## Supplementary Material

Additional file 1 **E-selectin expression in mouse dermal endothelial cells.** Sections were treated with anti-murine E-selectin and von Willebrand factor antibodies. E-selectin exhibits predominantly luminal **(A)** or intracellular **(B)** distribution in wild-type mouse skin. **(C)** shows E-selectin and **(D)** von Willebrand localisation as a marker of endothelial cells from a sdc-3 −/−mouse, **(E)** is a merge of C and D. Cell nuclei are blue using DAPI stain. Bar = 20 μm. Click here for file
