# Supplementary figures and images for: Syndecan-3 is selectively pro-inflammatory in the joint and contributes to antigen-induced arthritis in mice
Source: Arthritis Res Ther. 2014 Jul 11;16(4):R148. doi: 10.1186/ar4610 (PMC4227035; doi:10.1186/ar4610)

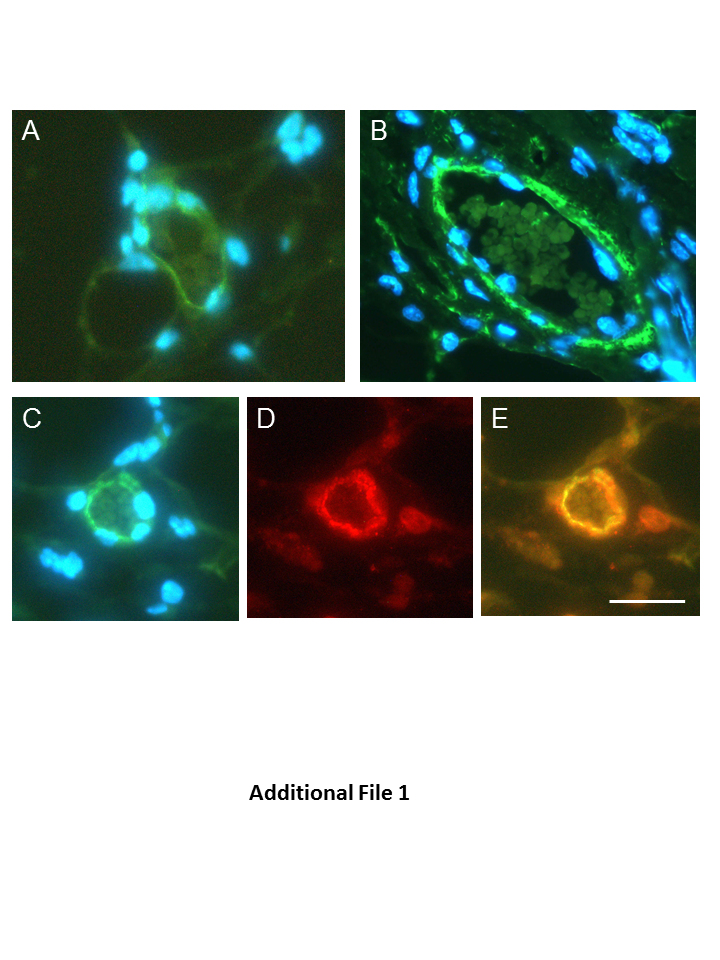

Supplement: Additional file 1 — E-selectin expression in mouse dermal endothelial cells. Sections were treated with anti-murine E-selectin and von Willebrand factor antibodies. E-selectin exhibits predominantly luminal (A) or intracellular (B) distribution in wild-type mouse skin. (C) shows E-selectin and (D) von Willebrand localisation as a marker of endothelial cells from a sdc-3 −/−mouse, (E) is a merge of C and D. Cell nuclei are blue using DAPI stain. Bar = 20 μm. [file ar4610-S1.tiff]
